# Supplementary material for: Engineering dimer mutants of human geranylgeranyl pyrophosphate synthase
Source: PLoS One. 2025 Jan 15;20(1):e0317437. doi: 10.1371/journal.pone.0317437 (PMC11734896; doi:10.1371/journal.pone.0317437)
Supplement: S1 File — (PDF) [file pone.0317437.s001.pdf]

wwPDB X-ray Structure Validation Summary Report (i)

Jul 26, 2024 – 12:40 PM EDT

PDB ID	:	9CSL
Title	:	Structure of mutant human geranylgeranyl pyrophosphate synthase (Y246D-C247L) in complex with isopentenyl pyrophosphate
Deposited on	:	2024-07-24
Resolution	:	2.10 Å(reported)

This wwPDB validation report is for manuscript review

For Manuscript Review

This is a wwPDB X-ray Structure Validation Summary Report.

This report is produced by the wwPDB biocuration pipeline after annotation of the structure.

We welcome your comments at *validation@mail.wwpdb.org*
A user guide is available at
https://www.wwpdb.org/validation/2017/XrayValidationReportHelp
with specific help available everywhere you see the (i) symbol.

The types of validation reports are described at
http://www.wwpdb.org/validation/2017/FAQs#types.

The following versions of software and data (see references (i)) were used in the production of this report:

MolProbity	:	4.02b-467
Mogul	:	1.8.5 (274361), CSD as541be (2020)
Xtriage (Phenix)	:	1.13
EDS	:	2.37.1
buster-report	:	1.1.7 (2018)
Percentile statistics	:	20191225.v01 (using entries in the PDB archive December 25th 2019)
Refmac	:	5.8.0158
CCP4	:	7.0.044 (Gargrove)
Ideal geometry (proteins)	:	Engh & Huber (2001)

1 Overall quality at a glance (i)

The following experimental techniques were used to determine the structure:
X-RAY DIFFRACTION

The reported resolution of this entry is 2.10 Å.

Percentile scores (ranging between 0-100) for global validation metrics of the entry are shown in the following graphic. The table shows the number of entries on which the scores are based.

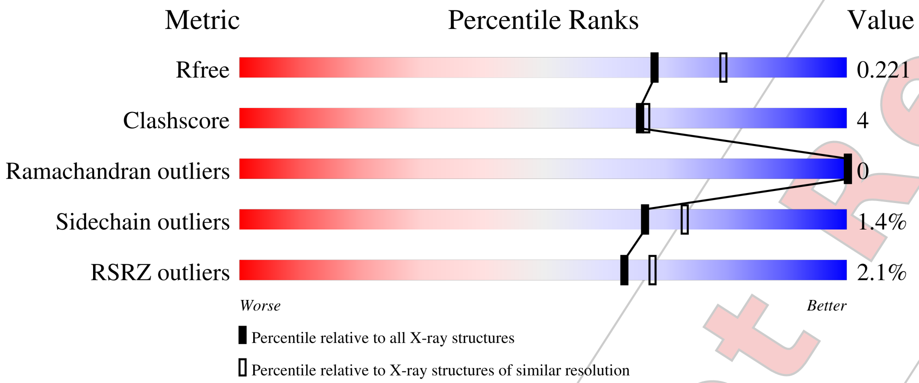

Metric	Whole archive (#Entries)	Similar resolution (#Entries, resolution range(Å))
R_{free}	130704	5197 (2.10-2.10)
Clashscore	141614	5710 (2.10-2.10)
Ramachandran outliers	138981	5647 (2.10-2.10)
Sidechain outliers	138945	5648 (2.10-2.10)
RSRZ outliers	127900	5083 (2.10-2.10)

The table below summarises the geometric issues observed across the polymeric chains and their fit to the electron density. The red, orange, yellow and green segments of the lower bar indicate the fraction of residues that contain outliers for >=3, 2, 1 and 0 types of geometric quality criteria respectively. A grey segment represents the fraction of residues that are not modelled. The numeric value for each fraction is indicated below the corresponding segment, with a dot representing fractions <=5% The upper red bar (where present) indicates the fraction of residues that have poor fit to the electron density. The numeric value is given above the bar.

Mol	Chain	Length	Quality of chain
1	A	322	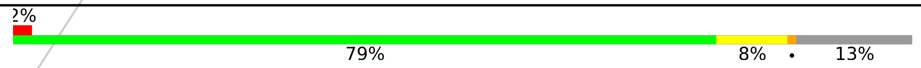
1	B	322	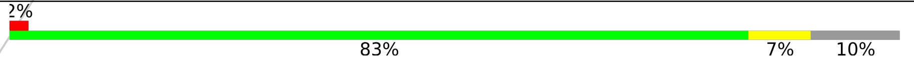

Ideal geometry (DNA, RNA) : Parkinson et al. (1996)
Validation Pipeline (wwPDB-VP) : 2.37.1

For Manuscript Review

2 Entry composition (i)

There are 5 unique types of molecules in this entry. The entry contains 5158 atoms, of which 0 are hydrogens and 0 are deuteriums.

In the tables below, the ZeroOcc column contains the number of atoms modelled with zero occupancy, the AltConf column contains the number of residues with at least one atom in alternate conformation and the Trace column contains the number of residues modelled with at most 2 atoms.

- Molecule 1 is a protein called Geranylgeranyl pyrophosphate synthase.

Mol	Chain	Residues	Atoms					ZeroOcc	AltConf	Trace
1	A	281	Total 2308	C 1479	N 392	O 431	S 6	0	5	0
1	B	291	Total 2351	C 1519	N 391	O 434	S 7	0	2	0

There are 48 discrepancies between the modelled and reference sequences:

Chain	Residue	Modelled	Actual	Comment	Reference
A	-21	MET	-	expression tag	UNP O95749
A	-20	HIS	-	expression tag	UNP O95749
A	-19	HIS	-	expression tag	UNP O95749
A	-18	HIS	-	expression tag	UNP O95749
A	-17	HIS	-	expression tag	UNP O95749
A	-16	HIS	-	expression tag	UNP O95749
A	-15	HIS	-	expression tag	UNP O95749
A	-14	SER	-	expression tag	UNP O95749
A	-13	SER	-	expression tag	UNP O95749
A	-12	GLY	-	expression tag	UNP O95749
A	-11	VAL	-	expression tag	UNP O95749
A	-10	ASP	-	expression tag	UNP O95749
A	-9	LEU	-	expression tag	UNP O95749
A	-8	GLY	-	expression tag	UNP O95749
A	-7	THR	-	expression tag	UNP O95749
A	-6	GLU	-	expression tag	UNP O95749
A	-5	ASN	-	expression tag	UNP O95749
A	-4	LEU	-	expression tag	UNP O95749
A	-3	TYR	-	expression tag	UNP O95749
A	-2	PHE	-	expression tag	UNP O95749
A	-1	GLN	-	expression tag	UNP O95749
A	0	SER	-	expression tag	UNP O95749
A	246	ASP	TYR	engineered mutation	UNP O95749
A	247	LEU	CYS	engineered mutation	UNP O95749
B	-21	MET	-	expression tag	UNP O95749

Continued on next page...

For Manuscript Review

Continued from previous page...

Chain	Residue	Modelled	Actual	Comment	Reference
B	-20	HIS	-	expression tag	UNP O95749
B	-19	HIS	-	expression tag	UNP O95749
B	-18	HIS	-	expression tag	UNP O95749
B	-17	HIS	-	expression tag	UNP O95749
B	-16	HIS	-	expression tag	UNP O95749
B	-15	HIS	-	expression tag	UNP O95749
B	-14	SER	-	expression tag	UNP O95749
B	-13	SER	-	expression tag	UNP O95749
B	-12	GLY	-	expression tag	UNP O95749
B	-11	VAL	-	expression tag	UNP O95749
B	-10	ASP	-	expression tag	UNP O95749
B	-9	LEU	-	expression tag	UNP O95749
B	-8	GLY	-	expression tag	UNP O95749
B	-7	THR	-	expression tag	UNP O95749
B	-6	GLU	-	expression tag	UNP O95749
B	-5	ASN	-	expression tag	UNP O95749
B	-4	LEU	-	expression tag	UNP O95749
B	-3	TYR	-	expression tag	UNP O95749
B	-2	PHE	-	expression tag	UNP O95749
B	-1	GLN	-	expression tag	UNP O95749
B	0	SER	-	expression tag	UNP O95749
B	246	ASP	TYR	engineered mutation	UNP O95749
B	247	LEU	CYS	engineered mutation	UNP O95749

- Molecule 2 is 3-METHYLBUT-3-ENYL TRIHYDROGEN DIPHOSPHATE (three-letter code: IPE) (formula: $C_5H_{12}O_7P_2$) (labeled as "Ligand of Interest" by depositor).

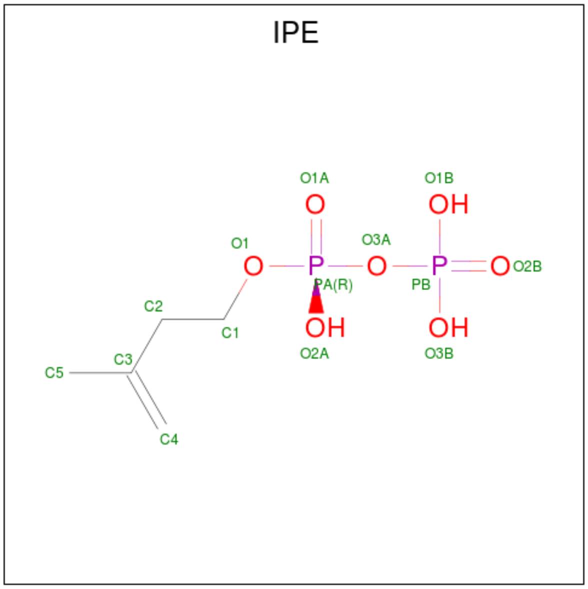

For Manuscript Review

Mol	Chain	Residues	Atoms				ZeroOcc	AltConf
			Total	C	O	P		
2	A	1	14	5	7	2	0	0

- Molecule 3 is CHLORIDE ION (three-letter code: CL) (formula: Cl).

Mol	Chain	Residues	Atoms		ZeroOcc	AltConf
			Total	Cl		
3	A	1	1	1	0	0

- Molecule 4 is SULFATE ION (three-letter code: SO4) (formula: O_4S).

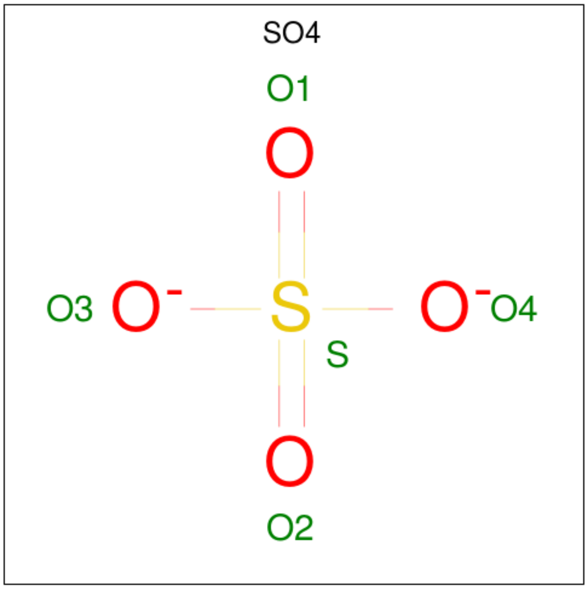

Mol	Chain	Residues	Atoms			ZeroOcc	AltConf
			Total	O	S		
4	A	1	5	4	1	0	0
4	A	1	5	4	1	0	0
4	A	1	5	4	1	0	0
4	A	1	5	4	1	0	0
4	A	1	5	4	1	0	0
4	B	1	5	4	1	0	0
4	B	1	5	4	1	0	0
4	B	1	5	4	1	0	0

Continued on next page...

For Manuscript Review

Continued from previous page...

Mol	Chain	Residues	Atoms	ZeroOcc	AltConf
4	B	1	Total O S<br>5 4 1	0	0
4	B	1	Total O S<br>5 4 1	0	0
4	B	1	Total O S<br>5 4 1	0	0
4	B	1	Total O S<br>5 4 1	0	0

- Molecule 5 is water.

Mol	Chain	Residues	Atoms	ZeroOcc	AltConf
5	A	230	Total O<br>230 230	0	0
5	B	194	Total O<br>194 194	0	0

For Manuscript Review

3 Residue-property plots (i)

These plots are drawn for all protein, RNA, DNA and oligosaccharide chains in the entry. The first graphic for a chain summarises the proportions of the various outlier classes displayed in the second graphic. The second graphic shows the sequence view annotated by issues in geometry and electron density. Residues are color-coded according to the number of geometric quality criteria for which they contain at least one outlier: green = 0, yellow = 1, orange = 2 and red = 3 or more. A red dot above a residue indicates a poor fit to the electron density (RSRZ > 2). Stretches of 2 or more consecutive residues without any outlier are shown as a green connector. Residues present in the sample, but not in the model, are shown in grey.

- Molecule 1: Geranylgeranyl pyrophosphate synthase

Chain A:

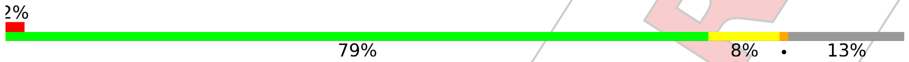

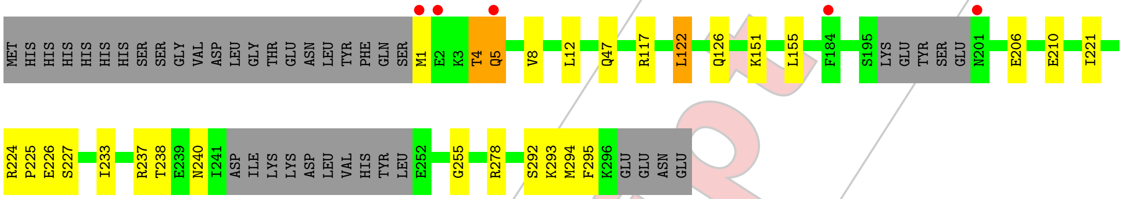

- Molecule 1: Geranylgeranyl pyrophosphate synthase

Chain B:

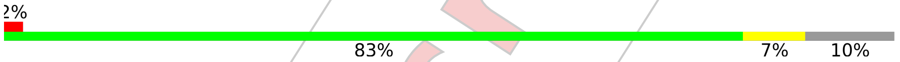

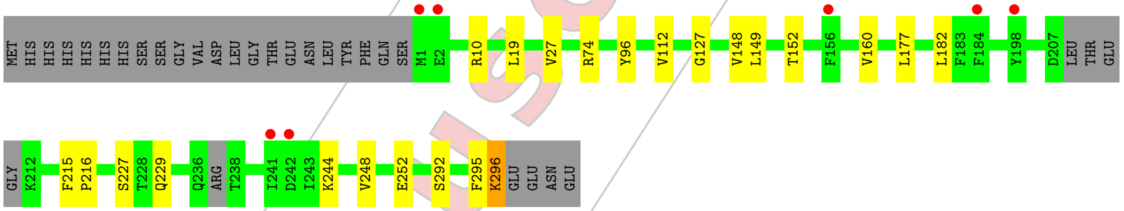

For Manuscript Review

4 Data and refinement statistics (i)

Property	Value	Source
Space group	P 6 2 2	Depositor
Cell constants<br>a, b, c, α, β, γ	191.11Å 191.11Å 96.63Å<br>90.00° 90.00° 120.00°	Depositor
Resolution (Å)	95.56 – 2.10<br>95.56 – 2.10	Depositor<br>EDS
% Data completeness<br>(in resolution range)	99.8 (95.56-2.10)<br>99.9 (95.56-2.10)	Depositor<br>EDS
R_{merge}	0.10	Depositor
R_{sym}	(Not available)	Depositor
$< I/\sigma(I) >$ [1]	1.72 (at 2.10Å)	Xtriage
Refinement program	REFMAC 5.8.0425	Depositor
R, R_{free}	0.178 , 0.216<br>0.187 , 0.221	Depositor<br>DCC
R_{free} test set	3000 reflections (4.94%)	wwPDB-VP
Wilson B-factor (Å^2)	43.5	Xtriage
Anisotropy	0.059	Xtriage
Bulk solvent k_{sol}(e/Å^3), B_{sol}(Å^2)	0.38 , 61.1	EDS
L-test for twinning[2]	$< \|L\| >$ = 0.51, $< L^2 >$ = 0.34	Xtriage
Estimated twinning fraction	No twinning to report.	Xtriage
F_o,F_c correlation	0.96	EDS
Total number of atoms	5158	wwPDB-VP
Average B, all atoms (Å^2)	52.0	wwPDB-VP

Xtriage's analysis on translational NCS is as follows: *The largest off-origin peak in the Patterson function is 2.58% of the height of the origin peak. No significant pseudotranslation is detected.*

[1] Intensities estimated from amplitudes.

[2] Theoretical values of $< |L| >$, $< L^2 >$ for acentric reflections are 0.5, 0.333 respectively for untwinned datasets, and 0.375, 0.2 for perfectly twinned datasets.

For Manuscript Review

5 Model quality (i)

5.1 Standard geometry (i)

Bond lengths and bond angles in the following residue types are not validated in this section: SO4, CL, IPE

The Z score for a bond length (or angle) is the number of standard deviations the observed value is removed from the expected value. A bond length (or angle) with $|Z| > 5$ is considered an outlier worth inspection. RMSZ is the root-mean-square of all Z scores of the bond lengths (or angles).

Mol	Chain	Bond lengths		Bond angles	
		RMSZ	#|Z| >5	RMSZ	#|Z| >5
1	A	0.51	0/2355	0.85	2/3185 (0.1%)
1	B	0.50	0/2402	0.86	0/3253
All	All	0.51	0/4757	0.85	2/6438 (0.0%)

There are no bond length outliers.

All (2) bond angle outliers are listed below:

Mol	Chain	Res	Type	Atoms	Z	Observed(^{o})	Ideal(^{o})
1	A	278	ARG	NE-CZ-NH1	-5.72	117.44	120.30
1	A	294	MET	CG-SD-CE	5.71	109.33	100.20

There are no chirality outliers.

There are no planarity outliers.

5.2 Too-close contacts (i)

In the following table, the Non-H and H(model) columns list the number of non-hydrogen atoms and hydrogen atoms in the chain respectively. The H(added) column lists the number of hydrogen atoms added and optimized by MolProbity. The Clashes column lists the number of clashes within the asymmetric unit, whereas Symm-Clashes lists symmetry-related clashes.

Mol	Chain	Non-H	H(model)	H(added)	Clashes	Symm-Clashes
1	A	2308	0	2280	20	0
1	B	2351	0	2306	18	0
2	A	14	0	9	0	0
3	A	1	0	0	0	0
4	A	25	0	0	1	0
4	B	35	0	0	1	0
5	A	230	0	0	6	0

Continued on next page...

For Manuscript Review

Continued from previous page...

Mol	Chain	Non-H	H(model)	H(added)	Clashes	Symm-Clashes
5	B	194	0	0	6	0
All	All	5158	0	4595	37	0

The all-atom clashscore is defined as the number of clashes found per 1000 atoms (including hydrogen atoms). The all-atom clashscore for this structure is 4.

The worst 5 of 37 close contacts within the same asymmetric unit are listed below, sorted by their clash magnitude.

Atom-1	Atom-2	Interatomic distance (Å)	Clash overlap (Å)
1:A:117:ARG:NH2	5:A:501:HOH:O	2.19	0.74
1:A:224:ARG:NE	5:A:505:HOH:O	2.26	0.69
1:B:252:GLU:OE2	5:B:501:HOH:O	2.13	0.66
1:B:27:VAL:HB	4:B:405:SO4:O2	1.97	0.64
1:A:221:ILE:O	1:A:227:SER:OG	2.20	0.60

There are no symmetry-related clashes.

5.3 Torsion angles (i)

5.3.1 Protein backbone (i)

In the following table, the Percentiles column shows the percent Ramachandran outliers of the chain as a percentile score with respect to all X-ray entries followed by that with respect to entries of similar resolution.

The Analysed column shows the number of residues for which the backbone conformation was analysed, and the total number of residues.

Mol	Chain	Analysed	Favoured	Allowed	Outliers	Percentiles	
1	A	280/322 (87%)	277 (99%)	3 (1%)	0	100	100
1	B	287/322 (89%)	284 (99%)	3 (1%)	0	100	100
All	All	567/644 (88%)	561 (99%)	6 (1%)	0	100	100

There are no Ramachandran outliers to report.

5.3.2 Protein sidechains (i)

In the following table, the Percentiles column shows the percent sidechain outliers of the chain as a percentile score with respect to all X-ray entries followed by that with respect to entries of similar

For Manuscript Review

resolution.

The Analysed column shows the number of residues for which the sidechain conformation was analysed, and the total number of residues.

Mol	Chain	Analysed	Rotameric	Outliers	Percentiles	
1	A	250/292 (86%)	244 (98%)	6 (2%)	49	53
1	B	250/292 (86%)	248 (99%)	2 (1%)	81	86
All	All	500/584 (86%)	492 (98%)	8 (2%)	67	69

5 of 8 residues with a non-rotameric sidechain are listed below:

Mol	Chain	Res	Type
1	B	296	LYS
1	B	19	LEU
1	A	122	LEU
1	A	47[B]	GLN
1	A	233	ILE

Sometimes sidechains can be flipped to improve hydrogen bonding and reduce clashes. All (5) such sidechains are listed below:

Mol	Chain	Res	Type
1	A	90	ASN
1	A	124	GLN
1	A	192	ASN
1	B	5	GLN
1	B	192	ASN

5.3.3 RNA (i)

There are no RNA molecules in this entry.

5.4 Non-standard residues in protein, DNA, RNA chains (i)

There are no non-standard protein/DNA/RNA residues in this entry.

5.5 Carbohydrates (i)

There are no monosaccharides in this entry.

For Manuscript Review

5.6 Ligand geometry (i)

Of 14 ligands modelled in this entry, 1 is monoatomic - leaving 13 for Mogul analysis.

In the following table, the Counts columns list the number of bonds (or angles) for which Mogul statistics could be retrieved, the number of bonds (or angles) that are observed in the model and the number of bonds (or angles) that are defined in the Chemical Component Dictionary. The Link column lists molecule types, if any, to which the group is linked. The Z score for a bond length (or angle) is the number of standard deviations the observed value is removed from the expected value. A bond length (or angle) with $|Z| > 2$ is considered an outlier worth inspection. RMSZ is the root-mean-square of all Z scores of the bond lengths (or angles).

Mol	Type	Chain	Res	Link	Bond lengths			Bond angles		
					Counts	RMSZ	$\#\|Z\| > 2$	Counts	RMSZ	$\#\|Z\| > 2$
4	SO4	A	407	-	4,4,4	0.35	0	6,6,6	0.07	0
4	SO4	B	405	-	4,4,4	0.28	0	6,6,6	0.22	0
4	SO4	B	407	-	4,4,4	0.26	0	6,6,6	0.16	0
4	SO4	B	401	-	4,4,4	0.32	0	6,6,6	0.14	0
4	SO4	A	403	-	4,4,4	0.28	0	6,6,6	0.10	0
2	IPE	A	401	-	11,13,13	0.52	0	15,19,19	1.67	4 (26%)
4	SO4	B	402	-	4,4,4	0.48	0	6,6,6	0.27	0
4	SO4	A	405	-	4,4,4	0.36	0	6,6,6	0.18	0
4	SO4	B	403	-	4,4,4	0.28	0	6,6,6	0.29	0
4	SO4	B	404	-	4,4,4	0.31	0	6,6,6	0.08	0
4	SO4	B	406	-	4,4,4	0.27	0	6,6,6	0.23	0
4	SO4	A	406	-	4,4,4	0.35	0	6,6,6	0.09	0
4	SO4	A	404	-	4,4,4	0.30	0	6,6,6	0.18	0

In the following table, the Chirals column lists the number of chiral outliers, the number of chiral centers analysed, the number of these observed in the model and the number defined in the Chemical Component Dictionary. Similar counts are reported in the Torsion and Rings columns. '-' means no outliers of that kind were identified.

Mol	Type	Chain	Res	Link	Chirals	Torsions	Rings
2	IPE	A	401	-	-	3/13/13/13	-

There are no bond length outliers.

All (4) bond angle outliers are listed below:

Mol	Chain	Res	Type	Atoms	Z	Observed(°)	Ideal(°)
2	A	401	IPE	PA-O3A-PB	-4.27	118.18	132.83
2	A	401	IPE	O1B-PB-O3A	2.91	114.38	104.64
2	A	401	IPE	O3A-PB-O2B	-2.25	98.69	111.19
2	A	401	IPE	O3B-PB-O2B	2.06	118.75	110.68

For Manuscript Review

There are no chirality outliers.

All (3) torsion outliers are listed below:

Mol	Chain	Res	Type	Atoms
2	A	401	IPE	C1-C2-C3-C4
2	A	401	IPE	C1-C2-C3-C5
2	A	401	IPE	O1-C1-C2-C3

There are no ring outliers.

2 monomers are involved in 2 short contacts:

Mol	Chain	Res	Type	Clashes	Symm-Clashes
4	B	405	SO4	1	0
4	A	405	SO4	1	0

The following is a two-dimensional graphical depiction of Mogul quality analysis of bond lengths, bond angles, torsion angles, and ring geometry for all instances of the Ligand of Interest. In addition, ligands with molecular weight > 250 and outliers as shown on the validation Tables will also be included. For torsion angles, if less then 5% of the Mogul distribution of torsion angles is within 10 degrees of the torsion angle in question, then that torsion angle is considered an outlier. Any bond that is central to one or more torsion angles identified as an outlier by Mogul will be highlighted in the graph. For rings, the root-mean-square deviation (RMSD) between the ring in question and similar rings identified by Mogul is calculated over all ring torsion angles. If the average RMSD is greater than 60 degrees and the minimal RMSD between the ring in question and any Mogul-identified rings is also greater than 60 degrees, then that ring is considered an outlier. The outliers are highlighted in purple. The color gray indicates Mogul did not find sufficient equivalents in the CSD to analyse the geometry.

For Manuscript Review

Ligand IPE A 401

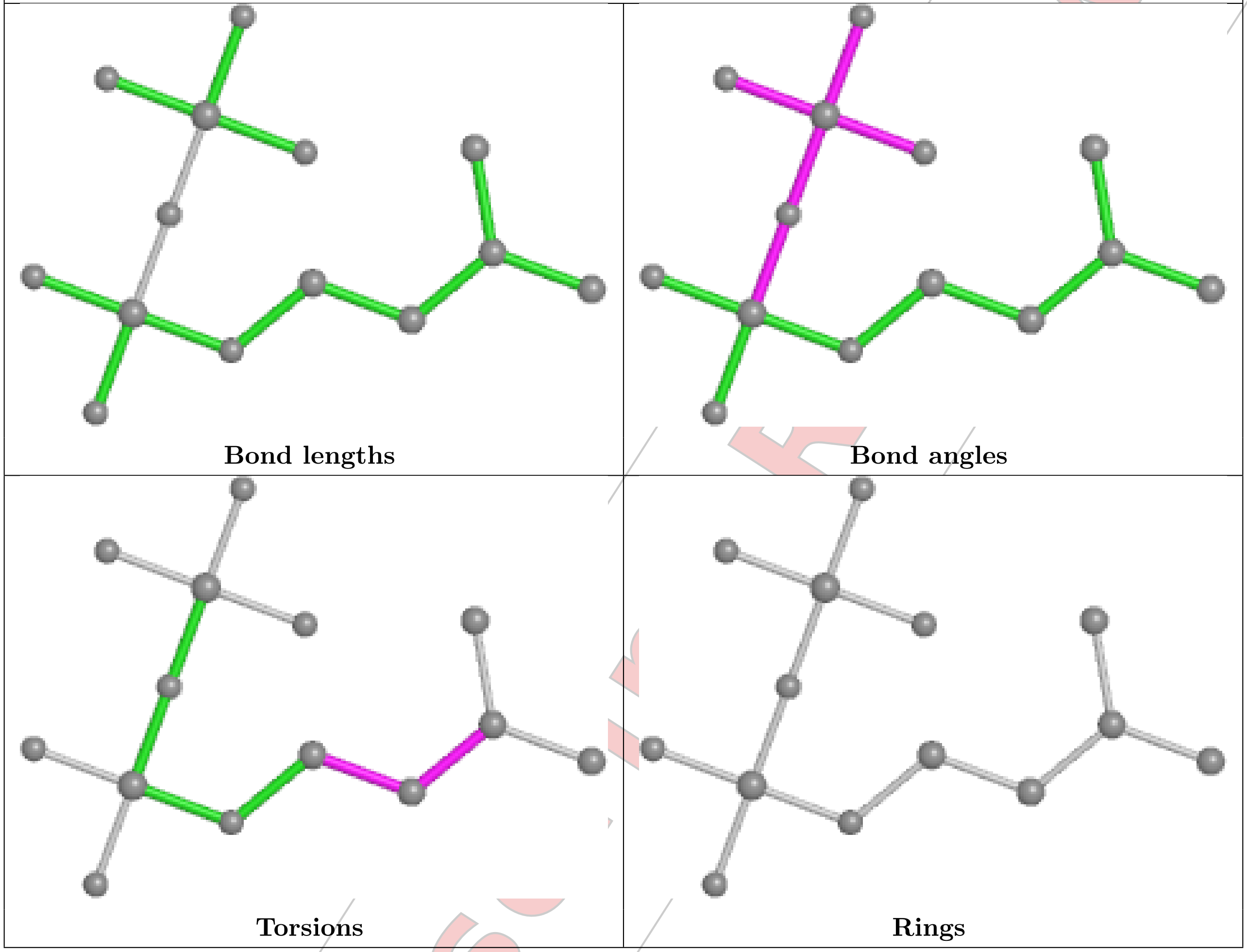

5.7 Other polymers (i)

There are no such residues in this entry.

5.8 Polymer linkage issues (i)

There are no chain breaks in this entry.

For Manuscript Review

6 Fit of model and data (i)

6.1 Protein, DNA and RNA chains (i)

In the following table, the column labelled '#RSRZ> 2' contains the number (and percentage) of RSRZ outliers, followed by percent RSRZ outliers for the chain as percentile scores relative to all X-ray entries and entries of similar resolution. The OWAB column contains the minimum, median, 95th percentile and maximum values of the occupancy-weighted average B-factor per residue. The column labelled 'Q< 0.9' lists the number of (and percentage) of residues with an average occupancy less than 0.9.

Mol	Chain	Analysed	<RSRZ>	#RSRZ>2			OWAB(Å^2)	Q<0.9
1	A	281/322 (87%)	-0.18	5 (1%)	68	72	32, 45, 81, 115	0
1	B	291/322 (90%)	-0.08	7 (2%)	59	64	34, 48, 90, 137	0
All	All	572/644 (88%)	-0.13	12 (2%)	63	68	32, 46, 90, 137	0

The worst 5 of 12 RSRZ outliers are listed below:

Mol	Chain	Res	Type	RSRZ
1	A	1	MET	3.4
1	A	201	ASN	3.3
1	A	184	PHE	2.7
1	B	1	MET	2.7
1	B	242	ASP	2.5

6.2 Non-standard residues in protein, DNA, RNA chains (i)

There are no non-standard protein/DNA/RNA residues in this entry.

6.3 Carbohydrates (i)

There are no monosaccharides in this entry.

6.4 Ligands (i)

In the following table, the Atoms column lists the number of modelled atoms in the group and the number defined in the chemical component dictionary. The B-factors column lists the minimum, median, 95th percentile and maximum values of B factors of atoms in the group. The column labelled 'Q< 0.9' lists the number of atoms with occupancy less than 0.9.

For Manuscript Review

Mol	Type	Chain	Res	Atoms	RSCC	RSR	B-factors(Å^2)	Q<0.9
4	SO4	A	407	5/5	0.65	0.28	137,144,149,160	0
4	SO4	B	407	5/5	0.79	0.24	75,97,102,121	0
4	SO4	B	404	5/5	0.85	0.36	96,108,115,119	0
4	SO4	B	405	5/5	0.85	0.19	82,84,118,120	0
4	SO4	B	406	5/5	0.85	0.21	98,98,122,126	0
4	SO4	A	406	5/5	0.85	0.34	106,113,124,127	0
2	IPE	A	401	14/14	0.91	0.20	57,79,92,93	0
4	SO4	A	403	5/5	0.94	0.21	90,90,95,101	0
4	SO4	B	401	5/5	0.94	0.22	91,93,98,115	0
3	CL	A	402	1/1	0.95	0.09	81,81,81,81	0
4	SO4	A	405	5/5	0.96	0.14	73,75,80,88	0
4	SO4	B	403	5/5	0.96	0.16	69,74,84,89	0
4	SO4	A	404	5/5	0.97	0.17	75,80,84,90	0
4	SO4	B	402	5/5	0.98	0.09	59,61,68,69	0

The following is a graphical depiction of the model fit to experimental electron density of all instances of the Ligand of Interest. In addition, ligands with molecular weight > 250 and outliers as shown on the geometry validation Tables will also be included. Each fit is shown from different orientation to approximate a three-dimensional view.

Electron density around IPE A 401:

2mF$_o$-DF$_c$ (at 0.7 rmsd) in gray
mF$_o$-DF$_c$ (at 3 rmsd) in purple (negative) and green (positive)

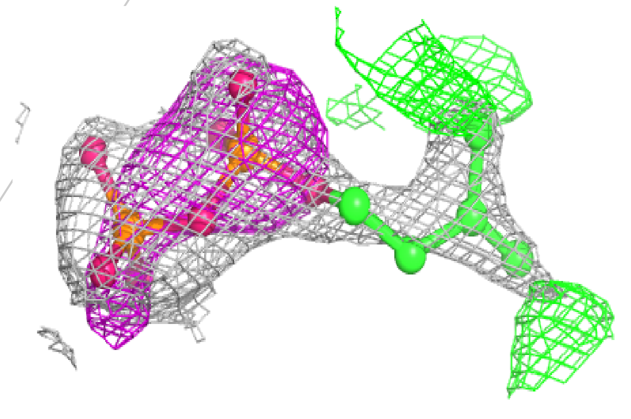

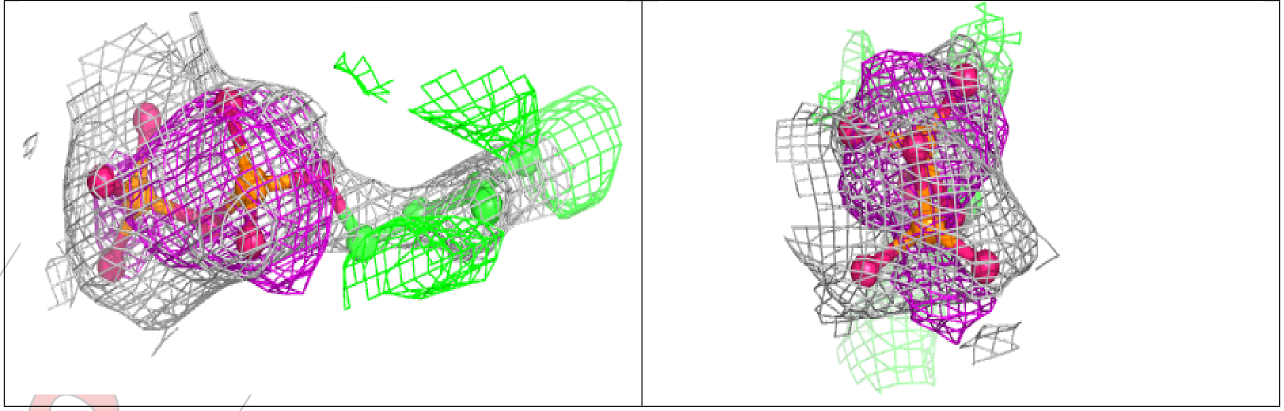

For Manuscript Review

6.5 Other polymers (i)

There are no such residues in this entry.

For Manuscript Review
