# Supplementary figures and images for: Engineering dimer mutants of human geranylgeranyl pyrophosphate synthase
Source: PLoS One. 2025 Jan 15;20(1):e0317437. doi: 10.1371/journal.pone.0317437 (PMC11734896; doi:10.1371/journal.pone.0317437)

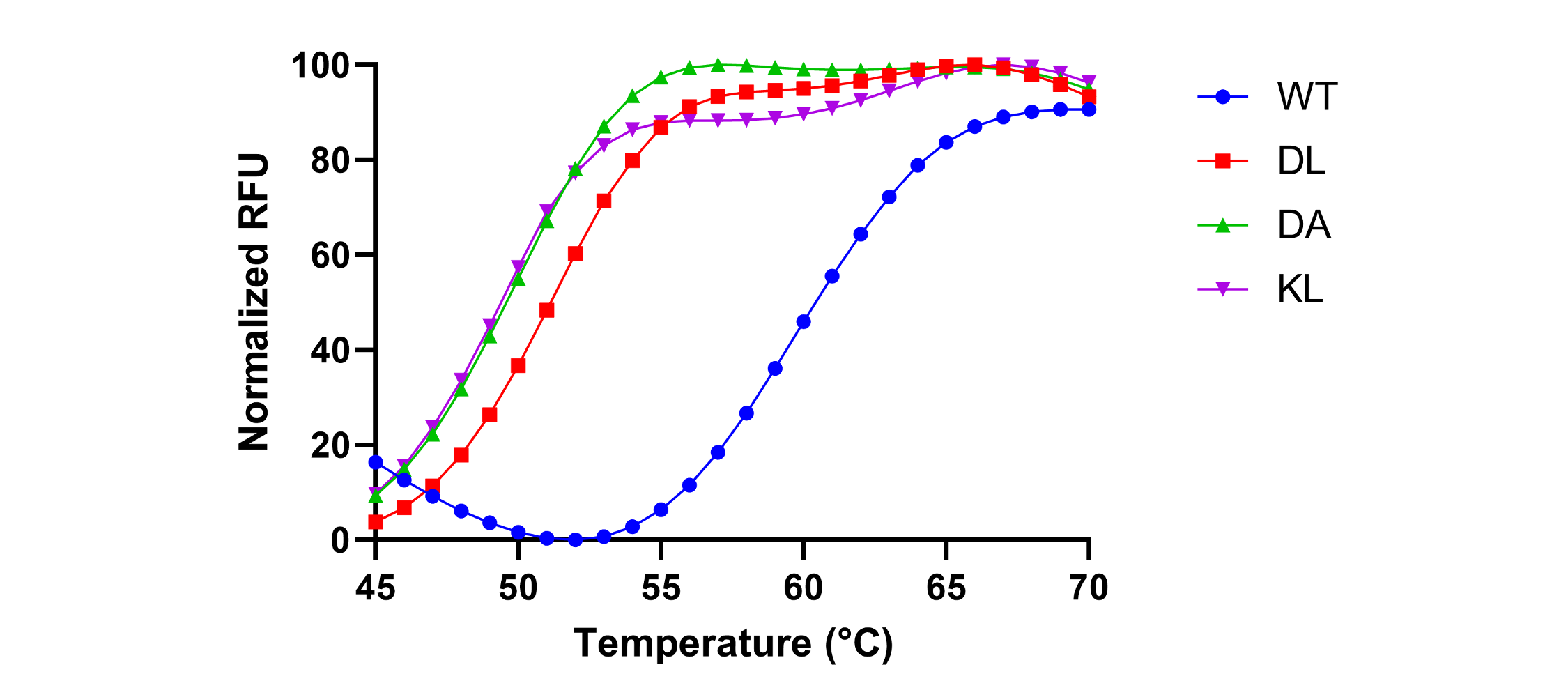

Supplement: S1 Fig — (TIF) [file pone.0317437.s002.tif]

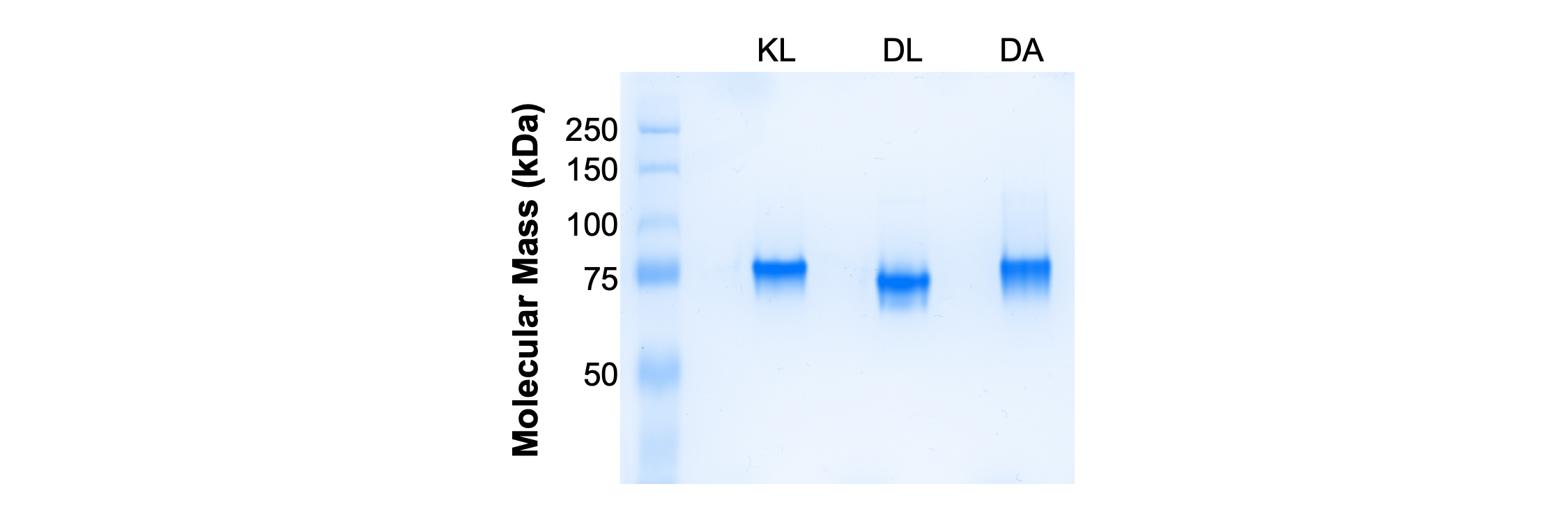

Supplement: S2 Fig — (TIF) [file pone.0317437.s003.tif]

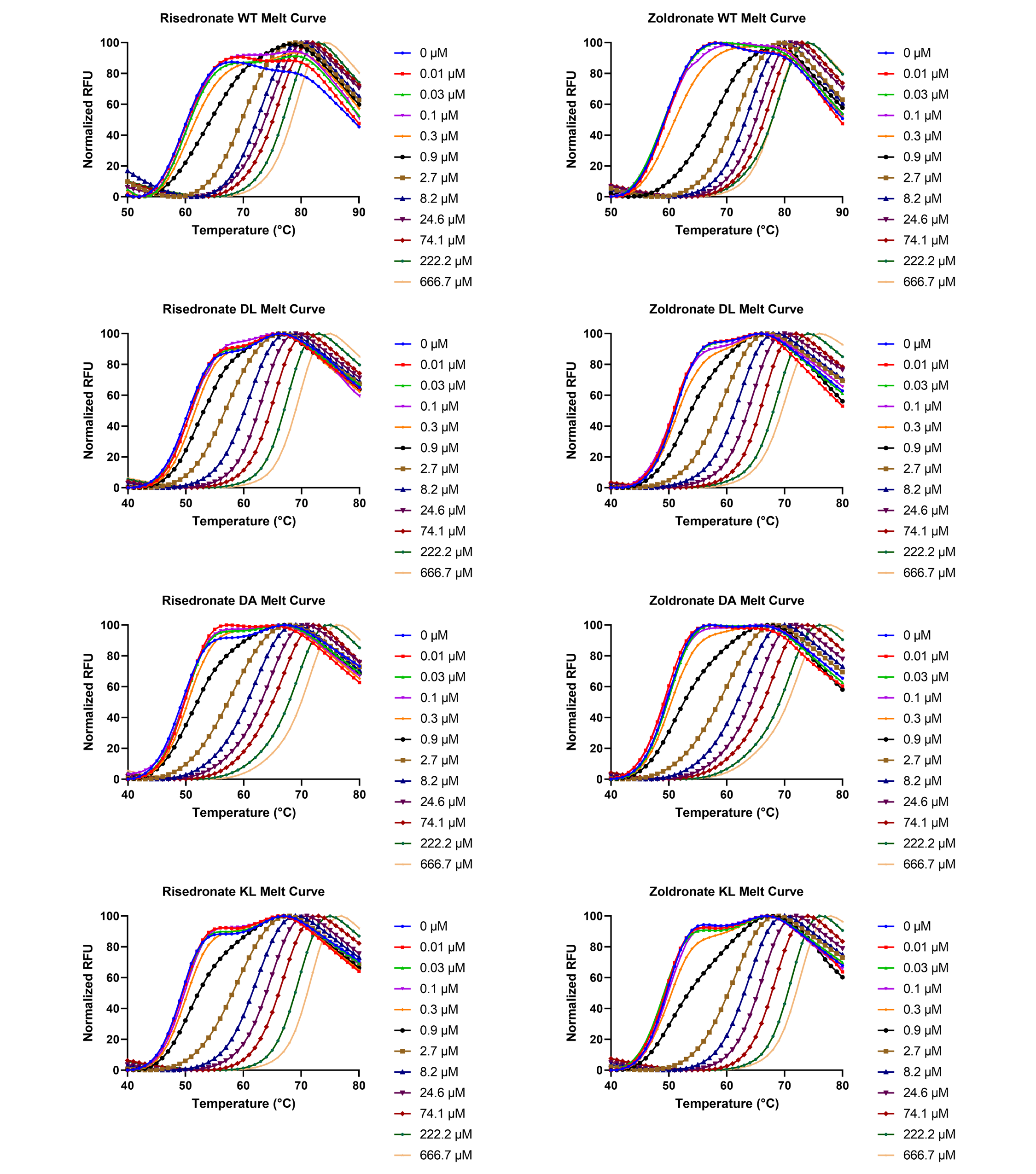

Supplement: S3 Fig — (TIF) [file pone.0317437.s004.tif]

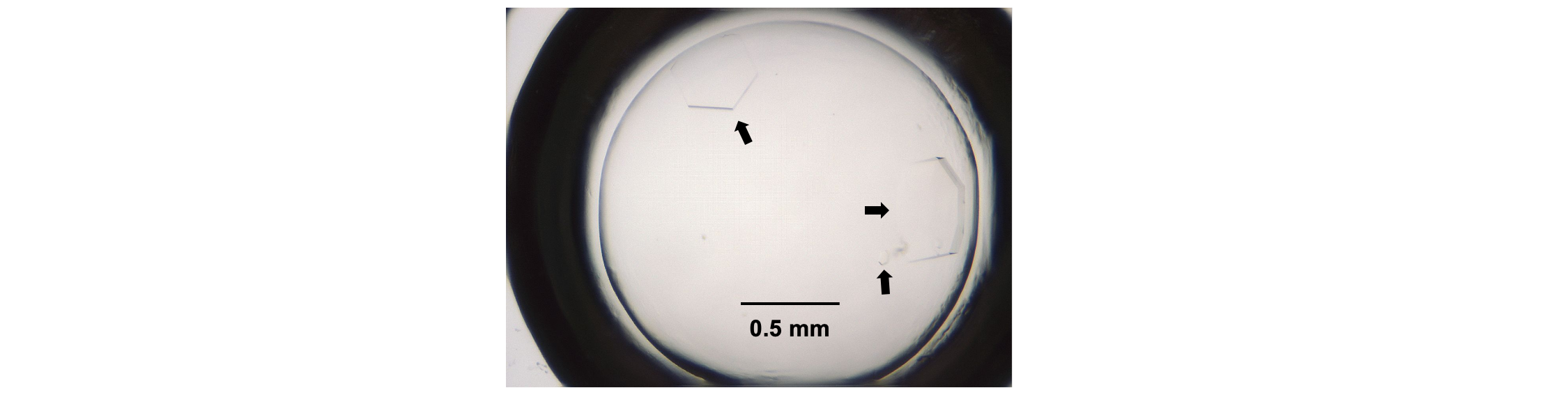

Supplement: S4 Fig — (TIF) [file pone.0317437.s005.tif]

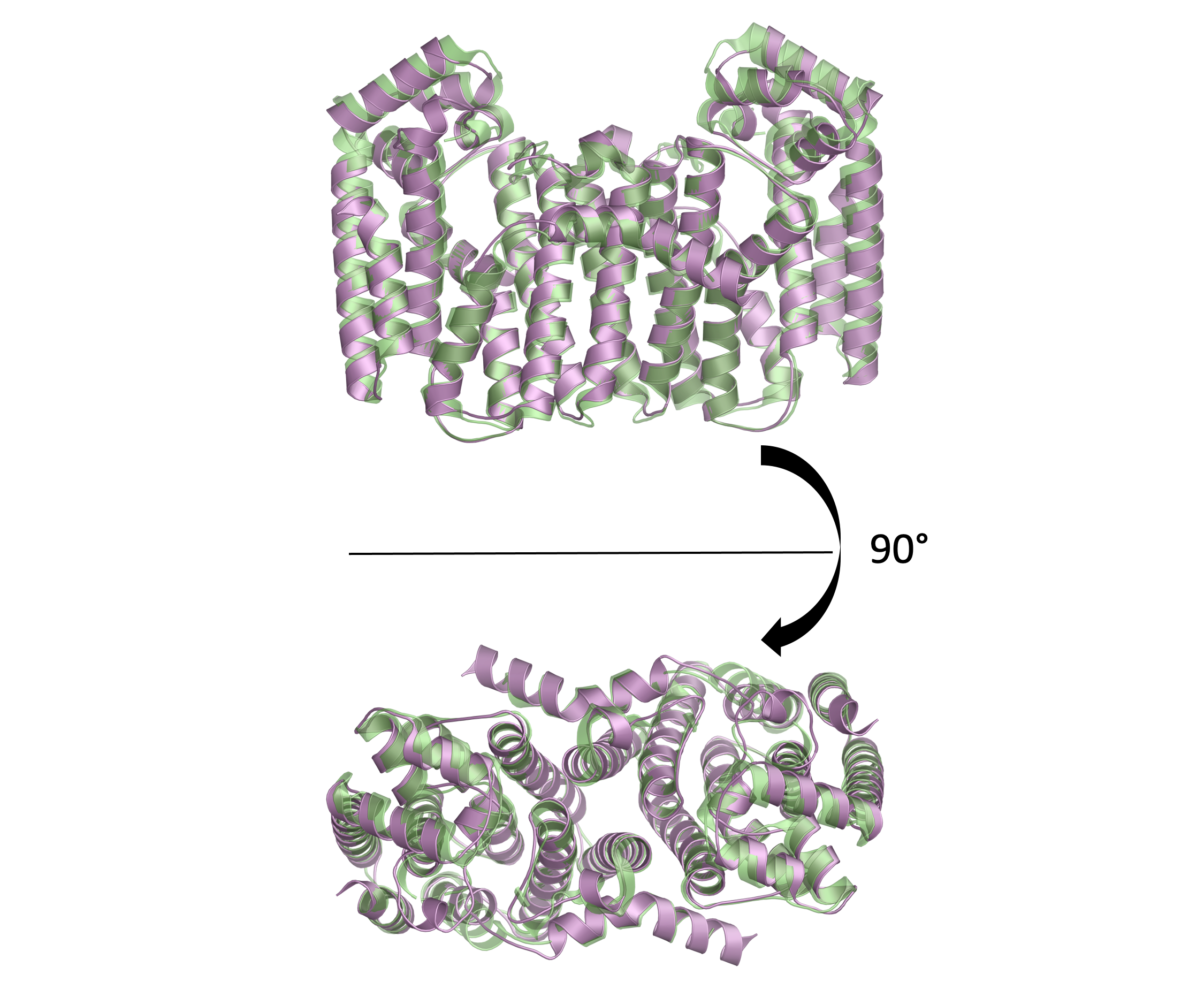

Supplement: S5 Fig — The AlphaFold-predicted DL mutant structure (purple) is superimposed onto the WT GGPPS structure (transparent green, PDB ID: 2Q80). The bottom image provides a top view of the same superposition. (TIF) [file pone.0317437.s006.tif]

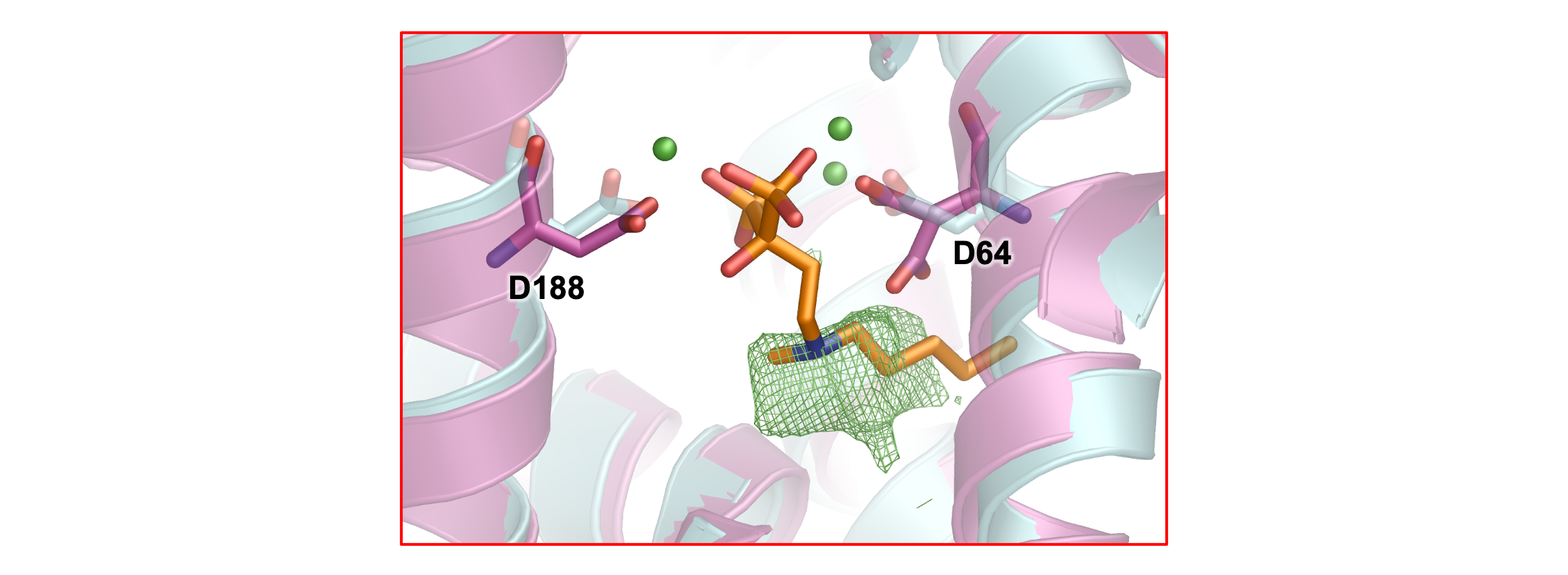

Supplement: S6 Fig — The DL mutant structure (magenta) is overlaid onto the ibandronate-bound WT GGPPS structure (semi-transparent cyan, ibandronate in orange; PDB ID: 6R4V). The green mesh represents the Fo − Fc difference map, contoured at 3 σ. Green spheres indicate magnesium ions co-bound with the bisphosphonate. The unidentified density overlaps with the position of the bisphosphonate side chain. Two alternate conformations of Asp64 were identified in the DL mutant structure. (TIF) [file pone.0317437.s007.tif]

**(A)**

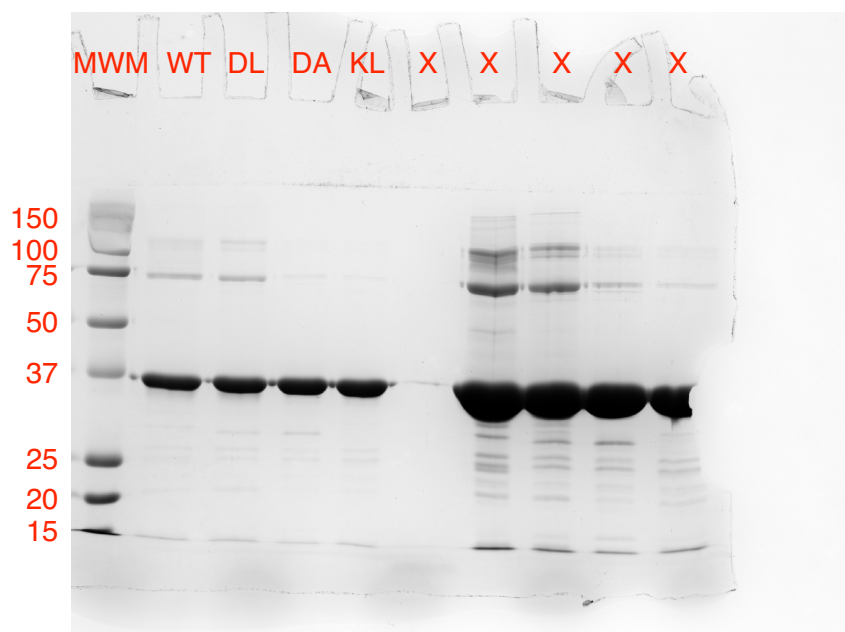

**(B)**

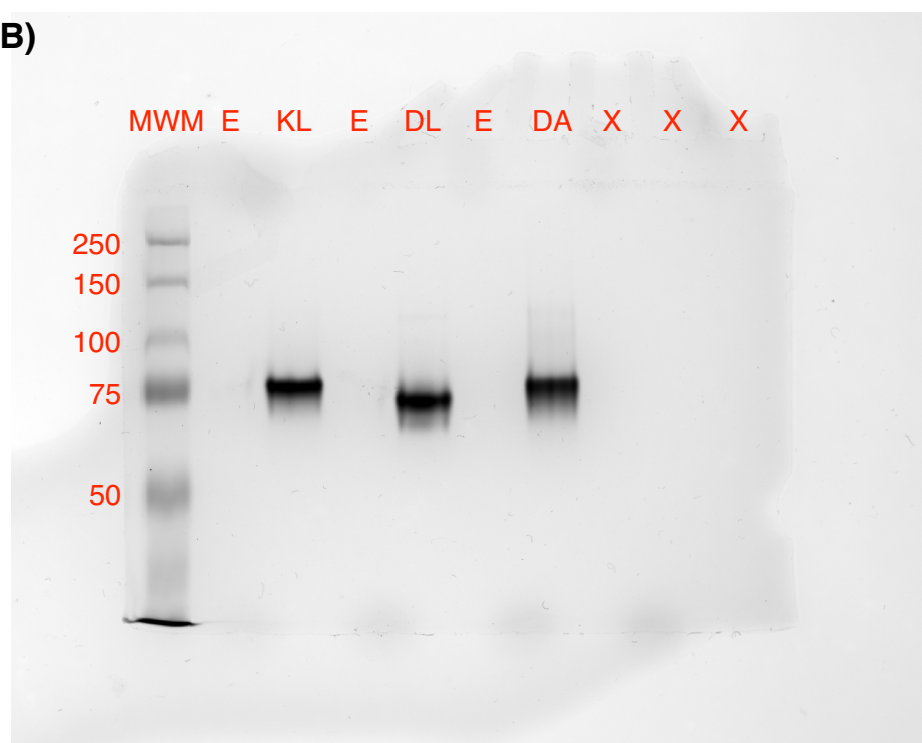

Supplement: S1 Raw images — (A) Original gel image for Fig 2B. Lanes loaded with WT and mutant protein samples are labeled. Lanes overloaded with identical samples and excluded from the final figure are marked with X. (B) Original gel image for S2 Fig. Lanes loaded with mutant protein samples are labeled, and empty lanes are marked with E. Empty lanes excluded from the final figure are marked with X. Both gels were imaged using a BioRad ChemiDoc Imaging System. (PDF) [file pone.0317437.s008.pdf]
